# New approach for efficient condensation heat transfer

**DOI:** 10.1093/nsr/nwy161

**Published:** 2018-12-22

**Authors:** Yaqi Cheng, Zuankai Wang

**Affiliations:** Department of Mechanical Engineering, City University of Hong Kong, China

The phase-change phenomena occurring at the solid, liquid and vapor triple interfaces, characterized by the multi-scale and multi-phase processes, are not only fundamentally important, but also relevant with multiple industrial applications including electronics cooling, water desalination, etc. [1]. Since phase-change processes are associated with large latent heat release, extensive efforts have been devoted to promoting or suppressing the phase change for a wide range of applications. Propelled by the advances and surge in nature-inspired interfacial surfaces over the past decade, it has been possible to rationally design materials and devices with tailored properties as well as to control interfacial phase-change transition to achieve increasingly higher energy efficiency. Indeed, several seminal papers underpinning the fundamentals of phase-change phenomena on textured surfaces are now among the top 15 sleeping beauty papers [2]. It is no exaggeration to say that a true Renaissance in the study of phase-change phenomena is now underway.

These phase-change processes are dramatically influenced by the geometrical and chemical properties of a surface. Conventional surfaces are limited by the capability to provide adequate nucleation sites, and at the same time delay the onset of the undesired liquid (in the case of condensation) or vapor (in the case of boiling) film. In the case of condensation, depending on the wettability of surfaces, the condensate droplets can exhibit filmwise or dropwise condensation mode. One of the long-standing challenges in condensation heat transfer is how to engineer surfaces to simultaneously enhance the multi-scale transport processes including droplet nucleation, growth and self-removal—all of which are subject to the trade-off imposed by individual intrinsic requirements [3]. While the use of hierarchical surfaces with hybrid wettability or slippery surfaces can decouple the conflicting requirements on rapid droplet nucleation and spontaneous droplet departure, the separation of these processes into different regions inherently undercuts the spatial advantages offered by textures [3,4]. To date, it remains challenging to achieve efficient condensation heat transfer in a wide spectrum of working environments.

**Figure 1.**
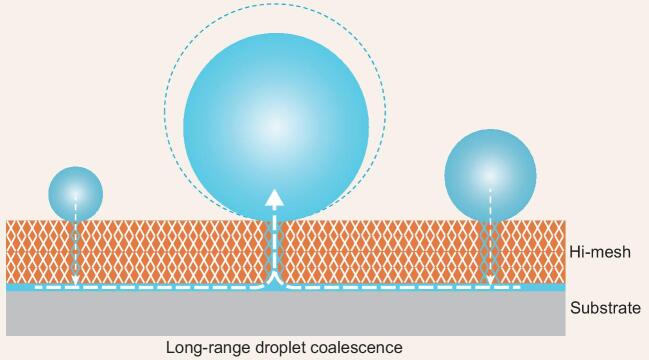
Schematic of long-range suction flow of condensation on porous surfaces consisting of the droplet-to-film coalescence and film-to-droplet suction flow for efficient surface refreshment and droplet growth.

Writing in the *National Science Review*, Wen *et al.* present a superhydrophobic hierarchical mesh-covered surface (hi-mesh) that exhibits an enhanced condensation heat transfer over a wide range of subcooling [5]. The authors observe a new suction flow condensation mode that realizes fast droplet growth and surface refreshment simultaneously. The secret lies in the formation of a spectacular liquid passage pathway sandwiched between the hi-mesh and copper plate, which serves to accelerate the draining of liquid film for pronounced surface refreshing, as shown in Fig. 1. Although the concept of the formation of a liquid channel for efficient condensation heat transfer has been demonstrated on gold nanowire arrays with hybrid wetting through long-range ensemble coalescence mechanism [6], this work by Wen *et al.* represents a step forward towards the realization of robust condensate surfaces for practical applications.

In spite of extensive progress, some issues remain to be overcome. Although the suction flow condensation yields a remarkable droplet growth rate that is much higher than those by direct steam condensation or droplet coalescence, the departure size of droplets is still at the scale of 1 mm, which is much larger than that of the self-jumping. Moreover, a more elegant approach should be proposed to tailor the geometry of the liquid channel, especially the thickness of the channel, in a well-controlled manner. One possible future direction is to integrate the hi-mesh with topological liquid diode that enables a rapid, long-range and directional transport of condensate liquid film without the need for any external energy input [7]. With further optimization, the surfaces reported in this work will find a wide range of applications such as in water and energy systems.
